# Overcoming the limits of piezoelectric composites

**DOI:** 10.1093/nsr/nwad205

**Published:** 2023-08-01

**Authors:** Ahmad Safari

**Affiliations:** Department of Materials Science and Engineering, Rutgers University, USA

The study of piezoelectric composites traces its origins back to the 1970s, when the concept of combining ceramics and polymers to introduce self-connectivity was first proposed [1–3]. Creating composites using polymers as the matrix and ferroelectric ceramics as fillers has proven to be an effective approach for improving the electromechanical coupling performance of piezoelectric materials. As flexible electronics have experienced significant advancements in recent years, the demand for high-performance flexible sensors, actuators and energy harvesters based on soft piezoelectric materials has also increased. This necessitates the development of piezoelectric composites that possess enhanced mechanical compliance and high piezoelectric coefficients.

However, achieving a balance between mechanical compliance and piezoelectric properties in polymer composites has posed a significant challenge for researchers. According to the effective media theory, improving polarization and piezoelectric performance in 0-3 type composites, despite their promising flexibility similar to the polymer matrix, remains a major challenge. Conversely, polymer composites filled with interconnected ceramic scaffolds, such as composites with 1-3 or 3-3 connectivity patterns, can yield much higher piezoelectric coefficients. Nevertheless, these composites require a high-volume fraction of ceramic fillers and exhibit limited flexibility when bent beyond a certain angle.

In their research, Tang *et al*. presented simulation results indicating that the lack of an efficient pathway for polarization and strain transfer in 0-3 composites significantly hampers the dielectric response of ceramic fillers, resulting in poor piezoelectric performance. Additionally, the calculated local electric field within the ferroelectric ceramic fillers, in 0-3 and 3-3 composites, is considerably smaller than that within the polymer matrix. This compromises the overall piezoelectric response of these composites [4].

To address the persistent challenge of balancing flexibility and piezoelectricity in polymer composites, Tang *et al*. put forth an innovative approach involving a composite with 3-3-3 connectivity patterns. In this novel configuration, the dielectric transition layer situated at the ceramic–polymer interface forms the third interconnected phase. Through the establishment of a micro-sized interconnected pathway within the lead zirconate titanate (PZT) ceramic scaffold, a robust transfer path for polarization and strain is created, resulting in ceramic–polymer composites with excellent flexibility and significantly enhanced piezoelectric performance. Additionally, the introduction of a tunable dielectric transition layer effectively homogenizes the electric field distribution within the composites, further improving their piezoelectric properties without compromising mechanical compliance. Remarkably, a highly flexible composite capable of enduring elastic deformations of up to 50%—similar to certain rubber materials—exhibited a remarkable piezoelectric coefficient of 250 pm V^−1^ and 120 pC N^−1^, which represents a significant breakthrough.

Undoubtedly, the concepts of 3-3-3 connectivity design and interface modulation in piezoelectric composites hold enormous potential for advancing the field of ‘soft’ electronics. This work is considered highly significant for researchers specializing in flexible piezoelectric materials, as well as those exploring various disciplines such as smart devices and soft robotics. The implications of these findings extend beyond a single field, making them relevant and valuable to a wide range of scientific communities.
